# A randomized trial of safety and pharmacodynamic interactions between a selective glucocorticoid receptor antagonist, PT150, and ethanol in healthy volunteers

**DOI:** 10.1038/s41598-021-88609-6

**Published:** 2021-05-10

**Authors:** Claire Morice, Dewleen G. Baker, Marguerite M. Patel, Tracy L. Nolen, Kayla Nowak, Shawn Hirsch, Thomas R. Kosten, Christopher D. Verrico

**Affiliations:** 1 Menninger Department of Psychiatry, Baylor College of Medicine, Houston, USA; 2 Michael E. DeBakey VA Medical Center, Houston, USA; 3 VA Center for Stress and Mental Health, VA San Diego Healthcare System, University of California, San Diego, San Diego, USA; 4 Social, Statistical and Environmental Sciences Unit, RTI International, Research Triangle Park, NC USA

**Keywords:** Drug safety, Pharmacodynamics, Drug development, Psychiatric disorders

## Abstract

PT150, a novel competitive glucocorticoid receptor (GR) antagonist, has proven safe in animal models, healthy volunteers, and people with depression. Our study is the first to investigate PT150’s safety with alcohol use. The primary objective of this study was to evaluate pharmacodynamic interactions between ethanol and PT150 in healthy subjects. This single-site, Phase I pilot trial consisted of community-recruited, healthy, alcohol-experienced participants aged 21–64 years. Of 32 participants screened, 11 were enrolled and randomized, one of which withdrew before intervention. PT150 (900 mg/day) was administered orally to all participants for five days. All participants received two beverage challenges on Day 1 (before PT150 administration) and Day 5 (after PT150 administration). On challenge days, they received both alcohol (16% ethanol) and placebo (1% ethanol) beverages in random order. Primary outcomes included breath alcohol level, blood pressure, heart rate, adverse events, and electrocardiogram changes. There were no statistically significant differences in vital signs or estimated blood alcohol concentrations between PT150 non-exposed and exposed groups during the ethanol challenge. There were no clinically significant abnormal electrocardiograms or serious adverse events. These data show that administration of PT150 with concurrent alcohol use is safe and well-tolerated. This study supports a future pharmacokinetic interaction study between PT150 and alcohol.

**Trial Registration** ClinicalTrials.gov Identifier: NCT03548714.

## Introduction

Glucocorticoid receptor (GR) antagonists have been investigated for numerous indications, including HIV^1,2^, metabolic syndrome^3,4^, and a myriad of psychiatric disorders. For example, mood disorders^5^, PTSD^6–8^, psychotic disorders^9^, and neurocognitive disorders^10,11^, which are all associated with hypothalamic–pituitary–adrenal (HPA) axis abnormalities, have garnered interest as potential therapeutic targets of GR antagonists. PT150 (previously Org 34517), a novel competitive GR antagonist, shows promise as a pharmacotherapeutic agent. PT150 is highly selective for GR, 100-fold more so than the non-selective GR and progesterone receptor antagonist mifepristone (RU486)^12^, a specificity that may allow PT150 to be a more effective treatment option with a more favorable side effect profile.

PT150 has exhibited a good safety profile in both animal models and human subjects, as demonstrated by 12 unpublished pre-clinical toxicology studies and 18 human studies that have been conducted to date. In mice, single dose toxicity studies of PT150, up to 2000 mg/kg, were well tolerated, and repeated dose toxicity studies conducted over 13 and 26 weeks showed good tolerability. Similarly, in single and repeated dose toxicokinetic studies in male cynomolgus monkeys, administration of PT150 (25 to 400 mg/kg/day) for 5 days was well tolerated. Six studies conducted in beagle dogs demonstrated no overt toxic effects, although one notable outcome was that liver microgranulomas developed in some beagles. However, the beagles recovered; the changes reverted to normal with cessation of treatment, and there was no biochemical evidence of hepatic damage. Of note, in a comparison study of PT150 and RU486 in beagles, both had a similar rate of liver microgranuloma formation. Reassuringly, neither RU486 nor PT150 is known to have inflammatory liver effects in humans. Regarding other safety outcomes, there is no evidence that PT150 impairs immunity: in an immunotoxicity study PT150 had no significant effects on the humoral (T-cell dependent antibody) immune response in rats. On the other hand, like RU486, PT150 has shown changes in reproductive organs in animals. In six out of seven reproductive toxicology studies performed in rats and rabbits, PT150 resulted in loss of pregnancy and teratogenicity but exhibited no evidence of maternal toxicity.

In humans, thirteen unpublished Phase I trials and five Phase II trials of PT150 have been conducted to date. An aggregate of the Phase I studies in healthy volunteers (n = 244) showed that PT150 was well tolerated in doses up to 900 mg daily over 14 days. No drug-related serious adverse events occurred. The most common drug-related adverse events were headache and nausea. The five Phase II trials conducted (protocol # 28104, 28105, 28106, 28130, and 28133) included a total of 704 patients with major depressive disorder (MDD) treated for 14 or 28 days. Of these patients, 428 received PT150 (118 on 150–300 mg/day, 212 on 300–600 mg/day, and 98 on 900 mg/day), 107 received paroxetine (10–40 mg/day), 9 received clomipramine (50–200 mg/day), and 160 received placebo. Two of these trials (protocol # 28130, 28133) studied participants who had MDD with psychotic features (n = 288). Expected adverse drug reactions included back pain, pruritis, hyperhidrosis, tremor, and cough. The incidence of adverse events (most commonly nausea, headache, and dizziness) for the PT150 groups was similar to that of the placebo groups. In the Phase II trials, 15 total serious adverse events (SAEs) occurred in the PT150 groups. 9 SAEs were deemed unlikely to be related or definitely not related to PT150 (cellulitis, urinary tract infection, pre-existing laryngeal cancer, and six hospitalizations for major depression, two of which included suicidal ideation). 3 SAEs were deemed possibly related to PT150 (all hospitalizations for major depression, one of which included psychotic features, and another which had a suicide attempt). Three SAEs were deemed probably related to PT150: two hospitalizations for major depression and one for a rash.

While PT150 has proven to be safe in healthy volunteers and in patients with MDD, it has yet to be investigated when concomitantly used with alcohol. The lifetime prevalence of alcohol consumption among U.S. adults is 86.3%^13^. Furthermore, given the nearly 30% lifetime prevalence of alcohol use disorder (AUD) in the general U.S. population, as well as AUD’s significant association with other mental health conditions^14^, it is critical to determine if PT150 is safe in individuals who consume alcohol. Because drug–drug interactions can cause profound clinical effects, either by reducing therapeutic efficacy or enhancing toxicity of drugs, we sought to determine the safety and tolerability of alcohol administration in subjects treated with PT150.

## Methods

### Study design

This was a phase I, single-site, within-subjects, drug–drug interaction study in non-treatment-seeking adults. The trial took place between September 11, 2018 and June 12, 2019 and was conducted at the Michael E. DeBakey VA Medical Center (MEDVAMC). Before initiation of the trial, the protocol was approved by the Baylor College of Medicine Institutional Review Board, MEDVAMC Research and Development Committee, US Army Medical Research and Development Command Human Research Protection Office, and Food and Drug Administration (IND#149591). The trial was also conducted in accordance with the Code of Federal Regulations and International Conference on Harmonization’s Guideline for Good Clinical Practice. No formal interim analyses were planned or conducted. An external independent data and safety monitoring committee oversaw the trial and monitored the results and the occurrence of adverse events. The trial was registered with clinicaltrials.gov, Identifier NCT03548714, 07/06/2018.

### Participant screening procedures

Healthy, alcohol-experienced participants aged 21–64 were recruited from the greater Houston metropolitan area via the Baylor College of Medicine clinical trials website, newspaper advertisements, flyers, and word of mouth. Interested individuals provided verbal consent prior to completing a pre-screening phone interview to determine if they met basic eligibility criteria. Basic eligibility criteria included interest in participating in a research study rather than a treatment study, being at least 21 years of age, not being pregnant or nursing, not taking medications or supplements at least 30 days prior to enrollment, and active alcohol use. Interested and eligible individuals were invited to complete an in-person interview at the VA. After providing written informed consent, potential subjects were excluded if they: displayed any latent signs of alcohol withdrawal per the Clinical Institute Withdrawal of Alcohol Assessment-Revised (CIWA-Ar)^15^, had a positive urine drug screen (except THC), a history of suicide attempts, suicidal ideation, substance use disorder, or major psychiatric disorder per the Mini International Neuropsychiatric Interview (MINI), a positive urine pregnancy test, indicated that they were nursing (females) or interested in fertility, a history of adrenal insufficiency per a self-reported medical history, or abnormal laboratory test results, which included an adrenocorticotropic hormone (ACTH) stimulation test, lipid panel, comprehensive metabolic panel, complete blood count, and thyroid stimulating hormone. The complete list of inclusion and exclusion criteria was published on clinicaltrials.gov. Eligible participants were enrolled and reported to the MEDVAMC inpatient ward within 30 days of the in-person interview to complete study procedures.

Before admission on day 1, a breathalyzer test, urine drug screen, and serum pregnancy test were conducted, and the CIWA-Ar was completed, ensuring the participant was still eligible. Subjects also completed the Timeline Follow Back (TLFB) for alcohol^16^ to evaluate the amount of alcohol used prior to the session.

### Alcohol administration sessions and outcome measures

Participants completed four alcohol challenge sessions: two on day 1 (pre-treatment) and two on day 5 (post-treatment, with PT150 at steady-state concentration). On both days 1 and 5, subjects consumed two beverages, a placebo beverage and an alcohol beverage containing 0.8 g/kg ethanol (approximately three standard drinks), administered in randomized order on day 1. Participants received one beverage at approximately 10 am, and the other beverage at least 4 h later, approximately 2 pm. Subjects were given 15 min to consume the beverage. The order of beverage consumption was reversed on day 5. Subjects received standardized meals to prevent confounding effects on alcohol levels.

The subjective measures used to assess responses to ethanol included the Alcohol Urge Questionnaire (AUQ), the Biphasic Alcohol Effects Scale (BAES), the Positive and Negative Affect Schedule (PANAS), and the Amphetamine, Morphine-Benzedrine, Lysergic Acid Diethylamide (LSD), Benzedrine, and the Pentobarbital-Chlorpromazine-Alcohol subscales from the Addiction Research Center Inventory (ARCI). The subjects completed these measures 30 min before each alcohol challenge session (i.e., baseline) and 15, 30, 60, 90, and 120 min after starting to consume the beverage.

The AUQ is an 8-item rating scale that consists of eight statements about the respondent’s feelings and thoughts about drinking as they are completing the questionnaire (i.e., right now). Subjects responded to each statement about alcohol craving, including desire to drink and an inability to avoid drinking if alcohol was available. Items were scored via a 7-item Likert scale ranging from “strongly disagree” to “strongly agree”, with higher scores reflecting greater craving^17^. The BAES is a 14-item adjective rating scale that is sensitive to the stimulant and sedative effects produced by ethanol^18^. Subjects indicated the extent to which they were feeling each adjective on a 11-point scale from “not at all” (0) to “extremely” (10). The stimulant scale score was measured by summing the scores for the adjectives elated, energized, excited, stimulated, talkative, up, and vigorous, and the sedation scale score was measured by summing the descriptors down, heavy head, sleepy, inactive, sedated, slow thoughts, and sluggish. The PANAS consists of two 10-item scales to measure both positive and negative affect. Subjects rated single-word items describing positive emotions (e.g., interested, excited, determined) or negative emotions (e.g., distressed, guilty, scared) on a scale from 1 (very slightly or not at all) to 5 (extremely). Scale scores range from 10 to 50, with higher scores indicating higher levels of positive or negative affect^19^. The ARCI is a standardized questionnaire that consists of 49 true/false statements^20^. The ARCI was specifically designed to be sensitive to the subjective effects of certain drugs or classes of drugs, including ethanol. This version of the ARCI consists of five empirically derived scales. For instance, the Pentobarbital-Chlorpromazine-Alcohol Group (PCAG) scale, which provides a measure of sedation, includes true/false statements such as “my speech is slurred” and “I am not as active as usual.” The Amphetamine (A) scale, which provide measures of stimulant-like effects, includes statements such as “my memory seems sharper to me than usual” and “I feel as if I could write for hours”. The Morphine–Benzedrine Group (MBG) scale, which reflects drug-induced euphoria, includes statements such as “I feel in complete harmony with the world and those about me”. The Lysergic Acid Diethylamide (LSD) scale, which reflects dysphoria/somatic effects, includes statements such as “my hand feels clumsy” and “I feel anxious and upset”. The ARCI has been used in numerous studies of ethanol and is both valid and reliable^21–23^.

The objective measures included heart rate, blood pressure, and estimated blood alcohol concentration (eBAC), which was obtained from breath using an Alco-Sensor IV (Intoximeter, St. Louis, MO). eBAC data were collected 15 min before each alcohol challenge session and 15, 30, 60, 105, and 120 min after starting to consume the beverage. Heart rate and blood pressure were collected 15 min before each alcohol challenge session and 5, 10, 15, 30, 45, 60, 75, 90, 105, and 120 min after starting to consume the beverage.

### Drugs

**Figure 1:**
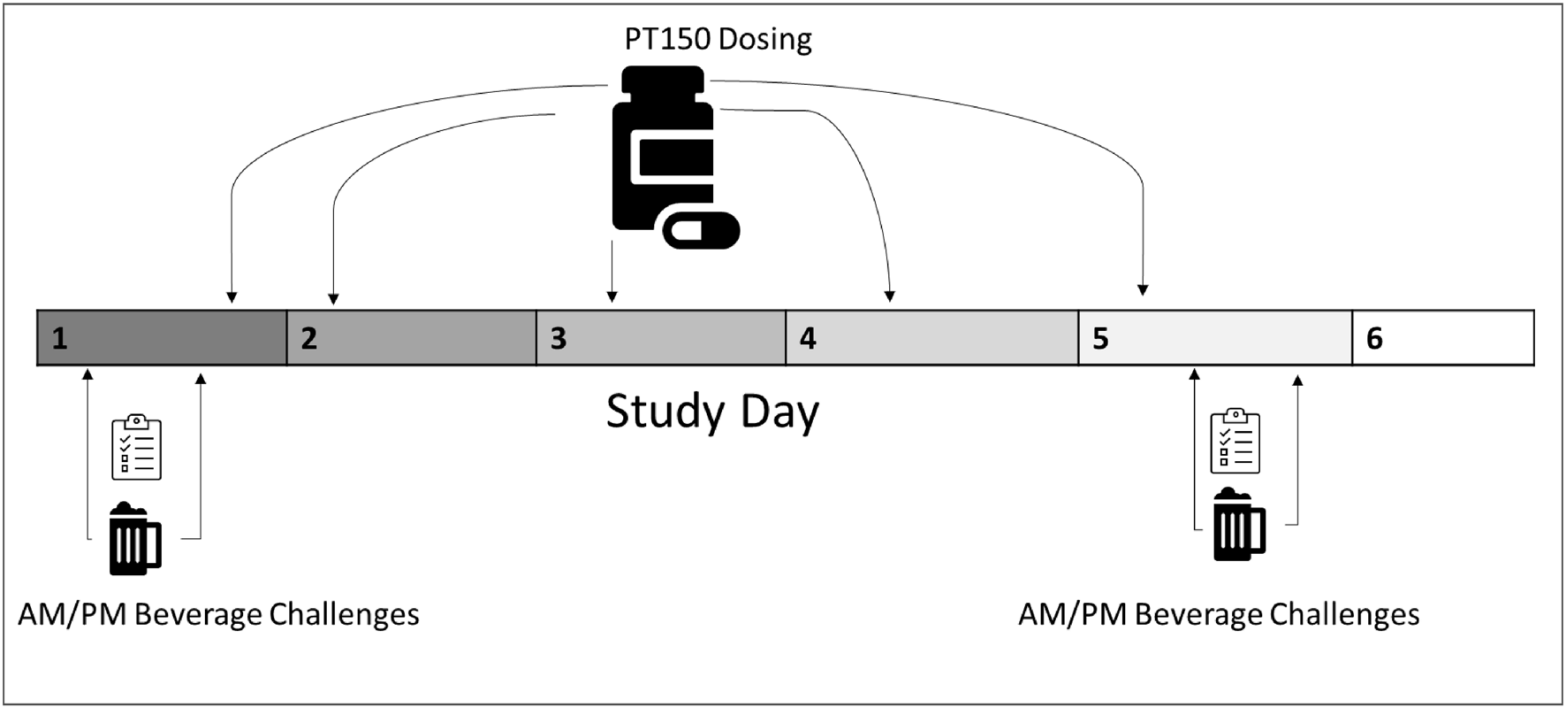
Study design.

As shown in Fig. 1, after both alcohol challenge sessions were completed on day 1, at approximately 5:00 PM, participants received the first PT150 dose (900 mg). Participants continued to receive PT150 once a day (900 mg, PO) at approximately 7:00 AM on days 2 through 5. The alcohol beverage was prepared with 95% ethanol (Everclear, St. Louis, MO) and grape Kool-Aid (Kraft Foods, Northfield, IL) to make up a 16% (0.8 g/kg dose) ethanol solution by volume. Placebo beverages consisted of the grape Kool-Aid plus 1% ethanol (0.05 g/kg dose) added to mask the taste. The order of beverage consumption was randomized using a permuted block design with randomly varying block sizes of 2 and 4. The beverages were prepared in bottles by the pharmacy and transported to the experimental rooms. Subsequently, staff poured the beverages from the bottles into cups with screw-top caps, which would prevent loss if the cup was tipped over. Cups contained a volume of 450 mL for a 70-kg person, with volume adjustments based on body weight. Because the density of Everclear alcohol (0.80 g/mL) is lower than Kool-Aid (1.02 g/mL), Everclear would float to the top of the beverage in between when the cups were filled by staff and when the drinks were consumed by the subjects. Subjects consumed the beverages from the cups by removing the caps, which allowed them to smell the floated alcohol in the placebo beverages and maintained blinding.

### Safety and daily measures

Prior to the first alcohol challenge session on day 1, participants completed an adverse events (AEs) symptoms checklist, had an electrocardiogram (ECG) conducted, and vital signs recorded to establish a baseline. The CIWA was completed on days 2–5 at approximately 7 AM while the AUQ, PANAS, BAES, and ARCI subscales were completed on days 2–4 at approximately 4 PM. On days 2–4, heart rate and blood pressure were collected twice, at approximately 7 AM and 12 PM, and eBACs were collected at approximately 7 AM and 3 PM. Heart rate and blood pressure were collected once on day 6, at approximately 7 AM. ECGs were conducted on days 2–6 at approximately 7 AM, and an additional ECG was conducted on day 1 one-hour post initial PT150 dosing. AE checklists were solicited/completed daily. Finally, blood was collected for laboratory assessments on days 3 (plasma cortisol and electrolytes) and 6 (plasma cortisol, electrolytes, thyroid stimulating hormone, and lipids).

### Statistical analysis

A sample size of 10 participants provided 80% power to detect an effect size of 0.85 for continuous safety measures (blood alcohol concentrations, subjective questionnaires, and vital signs) while receiving study drug and ethanol compared to ethanol alone using a one-sided paired t-test with α = 0.05 and assuming correlation of 0.5. The analysis was based on the safety population, defined as all participants that received any study drug, irrespective of amount or duration of treatment.

Changes in subjective questionnaire outcomes, heart rate, and blood pressure (from pre-challenge baseline collection) were analyzed using mixed effect models with study drug exposure (day 1 vs. 5), alcohol challenge (ethanol vs. placebo beverage), planned time as measured by minutes since beverage consumption, the pre-challenge value, and the two-way and three-way interactions between study drug, alcohol challenge, and time as fixed effects in the model. Each model included a random intercept and a variance component covariance pattern matrix for the within participants part of the model. Time was treated as a categorical variable so as to not assume linear trends over time. The models were used to generate difference in estimates and associated 95% confidence intervals for PT150 exposure vs. no exposure, alcohol vs. placebo beverage, and PT150 exposure vs. no exposure during the alcohol challenge. For eBAC, parameters of observed maximum concentration (C_max_), time of maximum concentration (T_max_), and area under the concentration curve at 2-h (AUC) calculated using the trapezoidal rule^24^ were analyzed. A similar model was run for these measures but without the time parameter.

Linear mixed effect models were used to compare post-baseline ECG and laboratory measurements to those obtained at baseline. Adverse event (AE) results were tabulated and summarized.

All statistical computations were performed and data summaries created using SAS 9.4. Presented p-values were based on two-sided tests. Due to the small sample size and exploratory nature of the study, p-values were not adjusted for multiplicity.

## Results

### Disposition and demographics

**Figure 2:**
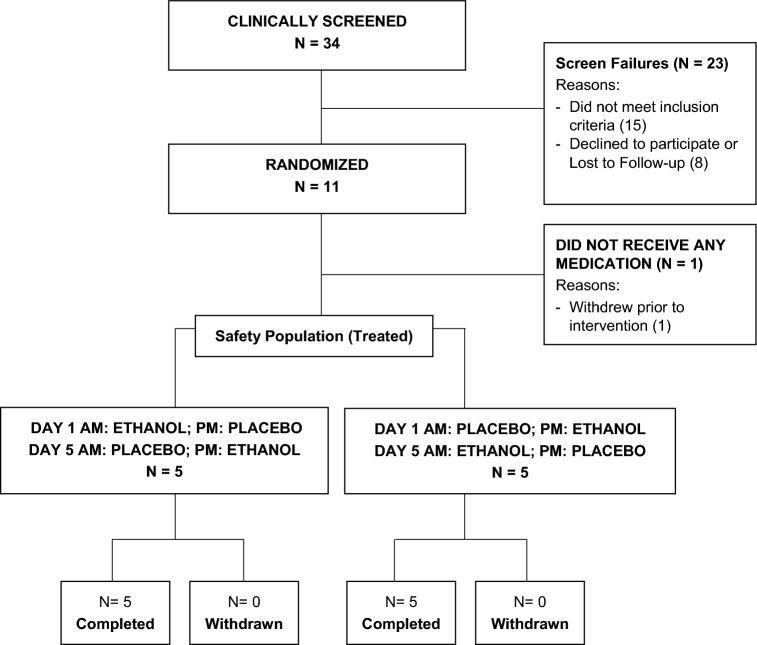
Study CONSORT.

A total of 34 volunteers were clinically screened, of which 11 met eligibility criteria and were randomized (Fig. 2). One randomized participant withdrew from the study on day 1 prior to first alcohol challenge session. The remaining ten participants completed all study procedures. These 10 participants represented the safety population.

The median age was 44.5 years, with a range of 28–63 years (Table 1). All 10 participants who completed the study were male. 70% were Black or African American and 20% reported being Hispanic or Latino. The median height was 179.1 cm, with a range of 155–199 cm, and the median weight was 80.9 kg, with a range of 68–136 kg.

**Table 1:** Summary of demographics.

Characteristic	Results
**Sex: n (%)**	
Female	0
Male	10 (100.0)
**Ethnicity: n (%)**	
Hispanic or Latino	2 (20.0)
Not Hispanic or Latino	7 (70.0)
Unknown	1 (10.0)
**Race: n (%)**	
Black or African American	7 (70.0)
White	3 (30.0)
Age (years): median (min–max)	44.5 (28–63)
Height (cm): median (min–max)	179.1 (155–191)
Weight (kg): median (min–max)	80.9 (68–136)

### Safety outcomes

**Figure 3:**
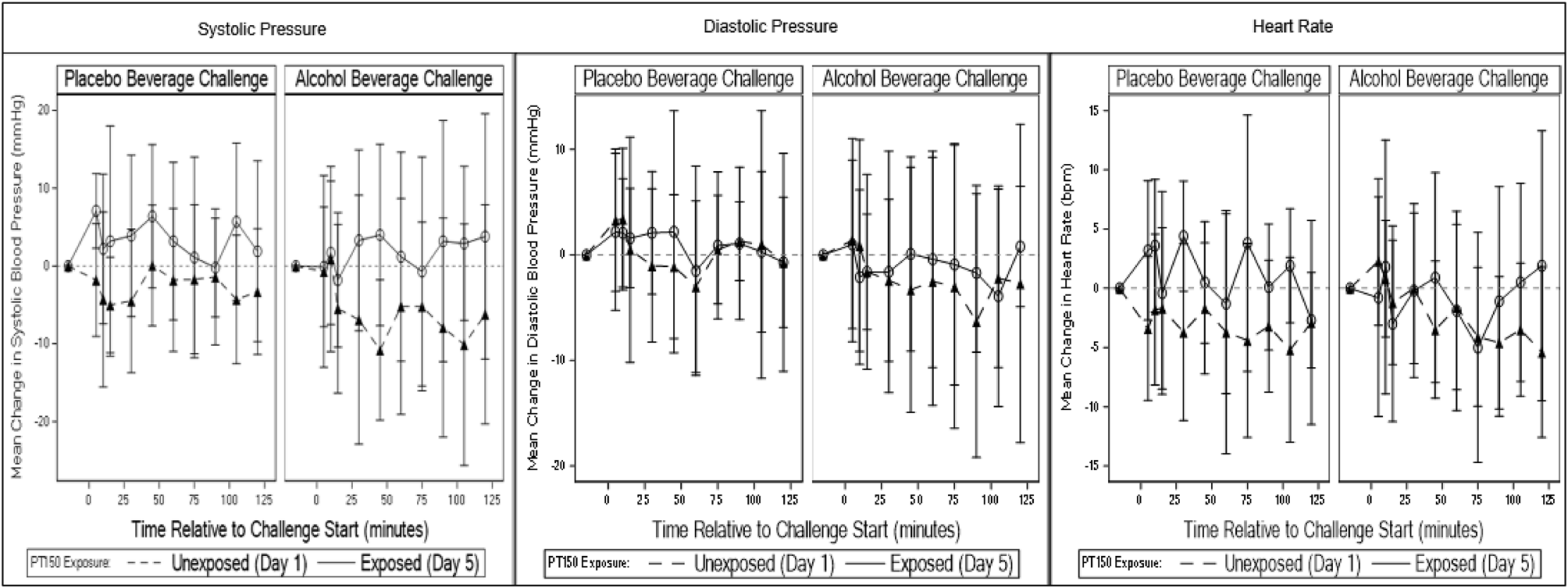
PT150 effects on physiological outcome measures; mean changes relative to the pre-challenge collection over the course of the placebo and alcohol challenges. Error bar represents ± 1 SD. Time 0 is the start of the challenge. The triangle symbol and dotted line represent Day 1 while the circle symbol and solid line represent Day 5.

For vital signs, there was no statistically significant evidence of a difference in estimated mean heart rate (difference (∆) = 0.57; 95% confidence interval (CI) − 1.01, 2.16; *p* = 0.48), systolic (∆ = 1.94; CI − 0.61, 4.49; *p* = 0.14), or diastolic (∆ = 0.50; CI − 1.41, 2.40; *p* = 0.61) blood pressure change from pre-challenge baseline between PT150 exposed (day 5) and non-exposed (day 1) during the ethanol challenge (Fig. 3). Similarly, no evidence of a difference was observed in change in these vital measures between overall PT150 exposed and non-PT150 exposed across challenge type (∆ = 0.32; CI − 0.85, 1.49; *p* = 0.60; ∆ = 0.83, CI − 1.12, 2.79, *p* = 0.40; ∆ = − 0.54, CI = − 1.90, 0.82, *p* = 0.44, respectively), nor between the ethanol and placebo challenges across PT150 exposure (∆ = 0.20, CI − 0.92, 1.32, *p* = 0.73; ∆ = − 0.10, CI − 1.83, 1.62, *p* = 0.91; ∆ = − 0.27, CI − 1.65, 1.11, *p* = 0.70, respectively). Additionally, these vital measures generally remained consistent across study day (Supplemental Figs. S1–S3).

**Figure 4:**
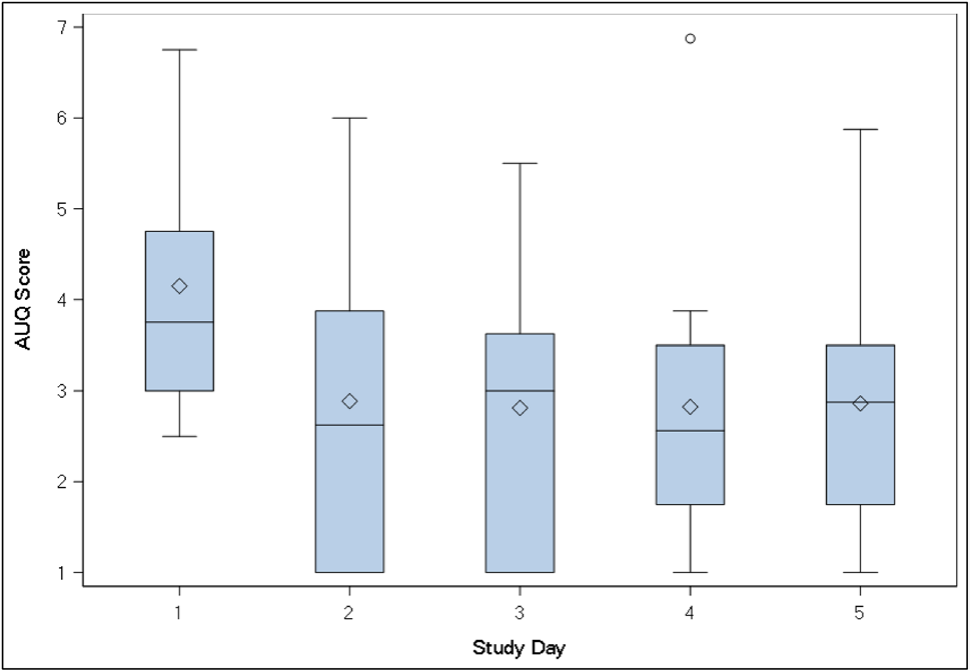
AUQ total score across study day; AUQ score is the average response across 8 questions (range 1:7). For comparability, the 09:30 AM collection was used for days 1 and 5. The box represents the Interquartile Range (IQR, 25th percentile to 75th percentile). Within the box, the horizontal line is the median and the symbol (triangle for day 1 and circle) is the mean. Whiskers represent the min/max, with the exception of outliers which fall outside the IQR by a distance greater than 1.5 times the IQR distance.

Subjective questionnaire outcome measures are summarized in Table 2. The average change in AUQ score decreased during the ethanol challenge without PT150 exposure (day 1) but increased over the ethanol challenge with PT150 at steady-state (day 5) (∆ = 1.30, CI 0.66, 1.95, *p* < 0.01). The change in AUQ score was also significantly lower for non-PT150 exposed compared to PT150 exposed irrespective of challenge type (∆ = 0.63, CI 0.19, 1.06, *p* < 0.01) and for the placebo challenge compared to the ethanol challenge irrespective of PT150 exposure (∆ = 0.49, CI 0.10, 0.88, *p* = 0.01). The observed differences in AUQ score change over the challenge appear to be due to higher pre-challenge means for non-PT150 exposed (day 1) compared to PT150 exposed (day 5). Non-challenge AUQ values were observed to decrease post-PT150 and remained relatively constant over the following study days (Fig. 4). While the average change in ARCI LSD scale did not differ during ethanol challenges between PT150 exposed and non-PT150 exposed, it was significantly higher overall for PT150 exposed (day 5) vs. non-exposed (day 1) irrespective of challenge type (∆ = 0.58; CI 0.06, 1.09, *p* = 0.03) and for the ethanol challenge compared to the placebo challenge irrespective of PT150 exposure (∆ = 0.64; CI 0.14, 1.14, *p* = 0.01). There was no evidence of ethanol or PT150 effect on any other subjective questionnaire outcome measures.

**Table 2:** PT150 effects on subjective questionnaire outcome measures.

Outcome measure	Adjusted mean estimate (standard error)				Comparisons estimated difference (95% CI), *P*^a^		
	Day 1		Day 5		PT150 vs control, EtOH challenge	PT150 vs control, overall	EtOH vs placebo, overall
	EtOH–CONTROL	Placebo–control	EtOH–PT150	Placebo–PT150			
AUQ	− 0.37 (0.30)	− 0.18 (0.29)	0.94 (0.30)	− 0.23 (0.28)	1.30 (0.66, 1.95); < **0.001**	0.63 (0.19, 1.06); **0.01**	0.49 (0.10, 0.88); **0.01**
**BAES**							
Stimulant	− 0.24 (2.89)	− 4.93 (2.86)	− 2.13 (2.86)	− 6.86 (2.86)	− 1.89 (− 8.96, 5.20); 0.60	− 1.91 (− 6.91, 3.09); 0.45	4.71 (− 0.28, 9.71); 0.06
Sedative	0.59 (2.10)	− 1.39 (2.14)	− 1.05 (2.13)	− 2.63 (2.12)	− 1.64 (− 5.77, 2.49); 0.44	− 1.44 (− 4.45, 1.58); 0.35	1.78 (− 1.12, 4.68); 0.23
**PANAS**							
Positive affect	− 1.33 (1.37)	− 1.70 (1.37)	− 0.96 (1.37)	− 1.24 (1.36)	0.37 (− 2.83, 3.57); 0.82	0.42 (− 1.85, 2.69); 0.72	0.33 (− 1.94, 2.59); 0.78
Negative affect	− 0.51 (0.59)	− 0.46 (0.55)	0.19 (0.57)	− 0.90 (0.55)	0.70 (− 0.60, 2.01); 0.29	0.13 (− 0.74, 1.00); 0.77	0.52 (− 0.31, 1.36); 0.22
**ARCI**							
LSD	− 0.29 (0.34)	− 0.94 (0.34)	0.28 (0.34)	− 0.36 (0.34)	0.57 (− 0.15, 1.29); 0.12	0.58 (0.06, 1.09); **0.03**	0.64 (0.14, 1.14); **0.01**
Pentobarbital	− 0.32 (0.66)	− 0.19 (0.66)	0.60 (0.66)	0.08 (0.66)	0.91 (− 0.69, 2.51); 0.26	0.59 (− 0.53, 1.70); 0.30	0.19 (− 0.92, 1.31); 0.73
Amphetamine	0.54 (0.59)	− 0.31 (0.55)	− 0.37 (0.55)	− 0.55 (0.55)	− 0.91 (− 2.48, 0.67); 0.26	− 0.57 (− 1.67, 0.53); 0.31	0.52 (− 0.58, 1.61); 0.36
Morphine	− 0.02 (0.94)	− 0.79 (0.94)	− 0.85 (0.94)	− 1.12 (0.94)	− 0.83 (− 3.34, 1.68); 0.51	− 0.58 (− 2.35, 1.19); 0.52	0.52 (− 1.25, 2.29); 0.56
Benzine	0.50 (0.55)	− 0.46 (0.52)	− 0.53 (0.53)	− 0.62 (0.52)	− 0.93 (− 2.40, 0.54); 0.22	− 0.54 (− 1.57, 0.48); 0.30	0.47 (− 0.55, 1.49); 0.36

^a^Mixed model used to determine if the mean change from baseline is significantly different between the Day 1 alcohol challenge (no PT150 exposure) and the Day 5 alcohol challenge (PT150 exposure), as well as between Day 1 (no PT150) and Day 5 (PT150), and Placebo and ethanol challenges.

Bolded values indicate statistically significant results at a *P* value ≤ 0.05.

**Figure 5:**
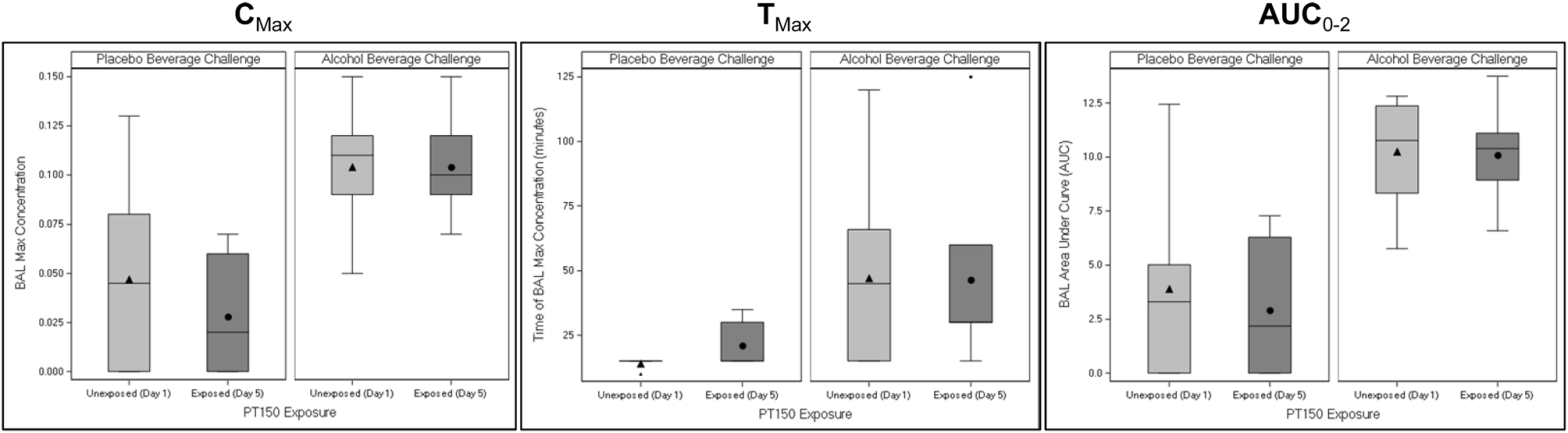
Pharmacokinetic effects of PT150 in combination with ethanol on blood alcohol levels as estimated from breathalyzer; the box represents the interquartile range (IQR, 25th percentile to 75th percentile). Within the box, the horizontal line is the median and the symbol (triangle for day 1 and circle) is the mean. Whiskers represent the min/max, with the exception of outliers which fall outside the IQR by a distance greater than 1.5 times the IQR distance.

For eBAC C_max_, T_max_, and AUC_0-2,_ there were no significant differences between PT150 exposed (day 5) and non-exposed (day 1) during the ethanol challenge (Fig. 5). The mean C_max_ for both PT150 exposed and non-exposed during the ethanol challenge was 0.104 (∆ = 0.00, CI − 0.03, 0.03, *p* = 0.99). T_max_ was also similar between PT150 exposed and non-exposed during the ethanol challenge (∆ = − 0.60, CI − 25.08, 23.89, *p* = 0.96). Last, AUC_0-2_ was also similar between PT150 exposed and non-exposed during the ethanol challenge (∆ = − 0.16, CI − 3.40, 3.08, *p* = 0.91).

One participant experienced a total of 5 mild AEs not related to study drug. One occurred on day 2, while four occurred on day 5 (Table 3). There were no clinically significant abnormal ECGs, Serious Adverse Events, or Unexpected Serious Adverse Reactions.

**Table 3:** Summary of adverse events by study day.

AE classification	# Events #Participants with event (% of participants with event)					
	Day 1 N = 10	Day 2 N = 10	Day 3 N = 10	Day 4 N = 10	Day 5 N = 10	Day 6 N = 10
**Any adverse events (AE)**						
Total	0 0 (100%)	1^a^ 1 (10%)	0 0 (100%)	0 0 (100%)	4^a^ 1 (10%)	0 0 (100%)
**AE severity**						
Mild	0 0 (100%)	1 1 (10%)	0 0 (100%)	0 0 (100%)	4 1 (10%)	0 0 (100%)
**AE relation to study drug**						
Definitely not related	0 0 (100%)	1 1 (10%)	0 0 (100%)	0 0 (100%)	4 1 (10%)	0 0 (100%)
**System organ class: MedDRA term**						
Eye disorders: eye pruritus	0 0 (100%)	0 0 (100%)	0 0 (100%)	0 0 (100%)	1 1 (10%)	0 0 (100%)
Eye disorders: lacrimation increased	0 0 (100%)	0 0 (100%)	0 0 (100%)	0 0 (100%)	1 1 (10%)	0 0 (100%)
Nervous system disorders: headache	0 0 (100%)	1 1 (10%)	0 0 (100%)	0 0 (100%)	0 0 (100%)	0 0 (100%)
Respiratory, thoracic and mediastinal disorders: nasal congestion	0 0 (100%)	0 0 (100%)	0 0 (100%)	0 0 (100%)	1 1 (10%)	0 0 (100%)
Respiratory, thoracic and mediastinal disorders: rhinorrhoea	0 0 (100%)	0 0 (100%)	0 0 (100%)	0 0 (100%)	1 1 (10%)	0 0 (100%)

^a^ The one AE experienced on day 2 and the four AEs experienced on day 5 all occurred on the same participant.

Mean plasma cortisol measurements were significantly higher on post-PT150 dosing days 3 and 6 compared to the pre-PT150 baseline plasma cortisol measurement (∆ = 5.01, CI 0.95, 9.06, *p* = 0.02; ∆ = 4.50, CI 0.45, 8.56, *p* = 0.03, respectively) (Table 4). All 10 participants had plasma cortisol levels in the normal range at baseline, while one was in the high range on day 3 and two were in the high range on day 6. Mean chloride levels at baseline were 102.40 mmol/L compared to 100.90 mmol/L on day 6 (∆ = − 1.50, CI − 3.00, 0.00, *p* = 0.05). Mean high-density lipoprotein (HDL) levels at baseline were 54.00 mmol/L compared to 46.20 mmol/L on day 6 (∆ = − 7.80, CI − 14.68, − 0.92, *p* = 0.03). There was no statistical evidence of a difference in mean sodium or potassium levels observed on day 3 or 6 compared to baseline or between the thyroid stimulating hormone, low-density lipoprotein, triglycerides, or total cholesterol levels observed at baseline, compared to day 6 (Table 4).

**Table 4:** PT150 effects on lab measurements.

Lab measurement	Adjusted mean estimates (SE) by visit		
	Baseline	Day 3	Day 6
Cortisol—mcg/dL	11.19 (1.59)	16.20 (1.59)^a^	15.69 (1.59)^b^
Sodium—mmol/L	135.50 (2.39)	139.75 (2.67)	137.50 (2.39)
Potassium—mmol/L	4.12 (0.13)	4.08 (0.13)	3.81 (0.13)
Chloride—mmol/L	102.40 (0.73)	101.64 (0.79)	100.90 (0.73)^b^
Thyroid stimulating hormone—mmol/L	1.25 (0.26)	N/D	1.53 (0.26)
Low-density lipoproteins—mmol/L	109.90 (11.39)	N/D	120.70 (11.39)
High-density lipoproteins—mmol/L	54.00 (3.49)	N/D	46.20 (3.49)^b^
Triglycerides—mmol/L	118.40 (15.27)	N/D	141.10 (15.27)
Total cholesterol—mmol/L	187.80 (13.27)	N/D	195.60 (13.27)

*N/D* not determined.

^a^Lab value collected on day 3 statistically different from the lab value collected on baseline based on *p* ≤ 0.05.

^b^Lab value collected on day 6 statistically different from the lab value collected on baseline based on *p* ≤ 0.05.

## Discussion

This Phase I pilot trial was the first to investigate the safety and tolerability of the glucocorticoid receptor antagonist PT150 with alcohol consumption. Overall, based on frequency of adverse events, physiological measures, and estimated pharmacokinetics, use of PT150 with alcohol was safe. There were no serious adverse events reported, and the five adverse events that occurred during alcohol use with PT150 (eye pruritis, increased lacrimation, headache, nasal congestion, and rhinorrhea) were reported by the same participant, rated mild, and deemed unrelated to the study drug. Physiology remained stable across all participants, as evidenced by no clinically significant changes in vital signs (heart rate, blood pressure) or electrolyte values between PT150 exposed and non-exposed. Statistically significant decreases in mean chloride levels and HDL levels were not deemed to be clinically significant, as final values were still within normal limits.

Given that heavy alcohol consumption and/or withdrawal is a risk factor for QTc prolongation^25–27^, P wave prolongation^28^, and ischemic heart disease mortality^29^, use of a cardiac-safe agent with co-occurring PT 150-alcohol use and evaluation of any drug–drug interaction effects on ECG are important. Although we observed a general decreasing trend in many ECG measures over time compared to the pre-PT150 baseline measure, changes were not deemed clinically significant.

These findings are in line with prior PT150 clinical trial outcomes. Collectively, data from the Phase I and Phase II studies showed no clinically relevant differences between treatment and control groups on cardiovascular measures (vital signs and ECGs) or laboratory results. ECG findings between the PT150 group (n = 428) and placebo group (n = 160) were similar: 0.5% in PT150 group with QT prolongation versus 0.6% in placebo group; 0.5% in PT150 group with other abnormal ECG findings versus 0.0% in placebo group; and 0.0% in PT150 group with other ECG changes versus 0.6% in the placebo group. Regarding other cardiac disorders, 0.2% exhibited left atrial dilatation compared to 0.0% in the placebo group, 0.5% experienced palpitations compared to 1.3% in the placebo group, 0.5% exhibited sinus tachycardia compared to 0.0% in the placebo group, and 0.5% exhibited tachycardia compared to 0.0% in the placebo group. None of the participants in the PT150 group developed myocardial ischemia or ventricular extrasystoles.

Prior glucocorticoid antagonist literature is also reassuring regarding cardiac safety. In an animal model for metabolic syndrome, obese and salt-sensitive rats who received mifepristone (RU486) actually demonstrated improvement in left ventricular fibrosis and oxidative stress as well as diastolic dysfunction^30^. Additionally, in a small (n = 20) randomized, double blind, placebo-controlled study, healthy men who received three total doses of oral mifepristone (1200 mg every 12 h) experienced no meaningful changes in QTc; the largest placebo-corrected, change-from-baseline QTc was 4.9 ms (90% CI 1.4–8.4), under the 10 ms cut-off for QTc effect^31^. In contrast, a prior thorough QT (TQT) study in which mifepristone 1800 mg was administered for 14 days showed a placebo-corrected, change-from-baseline QTc of 7.4 ms (90% CI 2.8–12.0)^32^. As noted above, human studies with PT150 alone revealed no significant group differences across any of the group ECG parameters. Our study is reassuring that PT150 did not cause clinically significant ECG changes at PT150 initiation or in response to increase in blood levels. Thus, while ECGs should be obtained and closely monitored as with any glucocorticoid antagonist, PT150 appears to be a safe compound when used with alcohol.

There was no evidence of a statistically significant difference in blood alcohol concentration summary measures with PT150 and ethanol compared to ethanol ingestion alone, including observed C_max_, T_max_, and AUC at 2-h. These results indicate that the eBAC do not change in the presence of PT150 at steady state, providing further evidence that alcohol can be safely consumed in the presence of PT150. Additionally, there were no statistically significant differences in subjective outcome measures, with the exception of measures of alcohol craving and subjective effects of alcohol intake, the AUQ and ARCI LSD scores. Averaged across the PT150 and non-PT150 exposure, the mean change in ARCI LSD score reduced 0.64 points more for alcohol alone compared to PT150 exposure at steady state, although this has no clear clinical implications. While the change in AUQ score reduced on average 1.30 points more for the alcohol-alone (day 1) compared to the PT150-steady state (day 5) condition across the alcohol challenges, this is likely not a clinically significant finding. Furthermore, this difference in AUQ score can be fully explained by the higher pre-challenge means for the alcohol alone condition (day 1) compared to PT150 steady-state condition (day 5). Although efficacy was not formally assessed as part of this phase I clinical trial, the fact that AUQ values were observed to decrease post-PT150 and subsequently remained relatively constant over the following study days suggests that PT150 may have a salutary effect on alcohol craving.

Lastly, mean plasma cortisol measurements were higher after PT150 exposure on days 3 and 6 compared to baseline on Day 1. This initial rise in cortisol levels is an anticipated outcome, as GR antagonism increases cortisol production by blocking negative feedback mechanisms of the HPA axis. Following cessation of treatment with a GR antagonist such as mifepristone, serum cortisol levels have been shown to return to even lower than baseline, effectively resetting the HPA axis^33^. Similarly, in a previous double-blind, placebo-controlled study (n = 274), PT150 induced a statistically significant dose-dependent increase in salivary cortisol levels after two weeks, which then normalized two weeks after active treatment ended (clinicaltrialsregister.eu, EudraCT 2004-002156-34)^34^. A longer trial is indicated to assess PT150’s long-term effects on cortisol levels and other neurohormone and endocrine biomarkers.

Our study is reassuring that PT150 can be used safely in patients who consume alcohol. Moreover, PT150 may even serve as a potential treatment for AUD, as supported by antiglucocorticoids’ modulation of AUD measures in preclinical and clinical studies. In murine models, suppression of corticosterone synthesis has been shown to decrease alcohol intake^35–37^, prevent alcohol-induced conditional learning^38^, and reduce memory loss in acute alcohol withdrawal^39^. In a clinical trial of people with alcohol dependence, the non-selective GR antagonist mifepristone (RU486) reduced alcohol cravings, decreased number of drinks per week, and improved liver-function markers without adverse events^40^. As previously mentioned, PT150’s greater selectivity for GRs may make it an even stronger and more targeted treatment option. In mice, PT150 reduced both the physiological severity and behavioral effects of alcohol withdrawal following binge-like ethanol administration without altering blood alcohol levels^41^. In short, PT150 may be an ideal way to selectively target GRs, safely improving AUD outcomes with minimal side effects.

In sum, PT150 has previously been shown to be well tolerated in Phase I and Phase II studies in subjects with no concomitant alcohol intake. Our study expands upon those findings by showing concurrent use of PT150 with alcohol to be well tolerated. One limitation of this study is that we used breath alcohol content rather than directly measuring blood alcohol concentration. Furthermore, this study does not address the use of PT150 during alcohol withdrawal, only with alcohol ingestion. Also, the all-male sample limits the generalizability of these findings to a broader population. Our study provides safety data for its application in an upcoming pharmacokinetic interaction study between PT150 and alcohol, as required by the FDA to move forward with a Phase II study of PT150 for treatment of co-occurring AUD and PTSD in Veterans. Based on laboratory studies, PT150 shows early evidence for antiviral (preliminary data) and anti-tumor activity (unpublished)^42–44^ as well. If these findings prove viable and development of PT150 for these applications in humans proceeds, these safety data alleviate concerns about PT150’s use in individuals across the general population, some of whom may be heavy alcohol users, as its use with alcohol consumption appears safe.

## Supplementary Information

Supplementary Information.
