# Supplementary material for: A randomized trial of safety and pharmacodynamic interactions between a selective glucocorticoid receptor antagonist, PT150, and ethanol in healthy volunteers
Source: Sci Rep. 2021 May 10;11:9876. doi: 10.1038/s41598-021-88609-6 (PMC8111026; doi:10.1038/s41598-021-88609-6)
Supplement: Supplementary file 1 — Supplementary Information. [file 41598_2021_88609_MOESM1_ESM.docx]

*A Randomized Trial of Safety & Pharmacodynamic Interactions Between a Selective Glucocorticoid Receptor Antagonist, PT150, and Ethanol in Healthy Volunteers*

*Claire Morice^1,2^, MD, Dewleen G. Baker^3^, MD, Marguerite M. Patel^1,2^, MD, Tracy L. Nolen^4^, DrPH, Kayla Nowak^4^, BS, Shawn Hirsch^4^, MPH, Thomas R. Kosten^1,2^, MD, Christopher D. Verrico^1,2^, PhD*

*^1^Baylor College of Medicine, Menninger Department of Psychiatry, ^2^Michael E. DeBakey VA Medical Center, ^3^University of California, San Diego, VA Center for Stress and Mental Health, VA San Diego Healthcare System, ^4^Social, Statistical and Environmental Sciences Unit, RTI International, Research Triangle Park, NC*

Corresponding author: Christopher D. Verrico, PhD; Email: [verrico@bcm.edu](mailto:verrico@bcm.edu)

Phone: 713-791-1414 x26849

Author contributions statements: DGB, TRK, and CDV designed the study. CDV, MMP, and TRK conducted the study. TLN, KN, and SH performed the statistical analysis.  CKM wrote the manuscript. All authors contributed to and approved the final manuscript.

*Abstract*:

**Background** PT150, a novel competitive glucocorticoid receptor (GR) antagonist, has proven safe in animal models, healthy volunteers, and people with depression. Our study is the first to investigate PT150’s safety with alcohol use.

**Objective** Evaluate for interactions between ethanol and PT150 in healthy subjects.

**Design, Setting, and Participants** Single-site Phase I pilot trial of community-recruited, healthy, alcohol-experienced participants aged 21-64 years. Of 32 participants screened, 11 were enrolled and randomized, one of which withdrew prior to intervention.

**Intervention** Five days of oral PT150 (900 mg/day) for all participants. All participants received two beverage challenges on Day 1 (prior to PT150 administration) and on Day 5 (after PT150 administration). On challenge days, they received both alcohol (16% ethanol) and placebo (1% ethanol) beverages in random order.

**Main Outcome Measures** Breath alcohol level; blood pressure; heart rate; adverse events; electrocardiogram changes.

**Results** There were no statistically significant differences in vital signs or estimated blood alcohol concentration between PT150 non-exposed and exposed groups during the ethanol challenge. There were no clinically significant abnormal electrocardiograms or serious adverse events.

**Conclusions** These data show PT150 with concurrent alcohol use is safe and well-tolerated. This study supports a future pharmacokinetic interaction study between PT150 and alcohol.

**Trial Registration** ClinicalTrials.gov Identifier: NCT03548714.

**Funding** Office of the Assistant Secretary of Defense for Health Affairs, Award No. W81XWH-15-2-0077

*Keywords:* Drug-drug interaction, Glucocorticoid Receptor Antagonist, hypothalamic-pituitary-adrenal axis, Alcohol Use Disorder

*Disclosures and declarations*: The U.S. Army Medical Research Acquisition Activity, 820 Chandler Street, Fort Detrick MD 21702-5014 is the awarding and administering acquisition office. This work was supported by the Office of the Assistant Secretary of Defense for Health Affairs through the Alcohol and Substance Abuse Research Program under Award No. W81XWH-15-2-0077. Opinions, interpretations, conclusions, and recommendations are those of the author and are not necessarily endorsed by the Department of Defense. The contents do not represent the views of the U.S. Department of Veterans Affairs or the United States Government.

*Acknowledgments:* This material is the result of work supported with resources and the use of facilities at the Michael E. DeBakey Veterans Affairs Medical Center in Houston, Texas.

**Supplemental Figure Set 1. Change in Physiological Outcomes by Study Day; includes the first vital sign collected on each study day. The box represents the Interquartile Range (IQR, 25th percentile to 75th percentile). Within the box, the horizontal line is the median and the diamond is the mean. Whiskers represent the min/max, with the exception of outliers which fall outside the IQR by a distance greater than 1.5 times the IQR distance.**

**Supplemental Figure 1. Heart Rate**

**Supplemental Figure 2. Systolic Blood Pressure**

**Supplemental Figure 3. Diastolic Blood Pressure**
